# CyberKnife Radiosurgery – Value as an Adjunct to Surgical Treatment of HCC?

**DOI:** 10.7759/cureus.591

**Published:** 2016-04-28

**Authors:** Markus Schoenberg, Andrey Khandoga, Sebastian Stintzing, Christoph Trumm, Tobias Simon Schiergens, Martin Angele, Mark op den Winkel, Jens Werner, Alexander Muacevic, Markus Rentsch

**Affiliations:** 1 Department of General, Visceral and Transplantation Surgery, Hospital of the University of Munich; 2 Surgery, Ludwig Maximilian University of Munich; 3 Internal Medicine, Ludwig Maximilian University of Munich; 4 Hospital of the University of Munich; 5 European Cyberknife Center Munich

**Keywords:** cyberknife, hcc, liver cancer, radiosurgery

## Abstract

**Introduction:**

CyberKnife radiosurgery (CK) is an effective tool for the treatment of malignancies. Its greatest potential is in high-dose radiosurgery delivered to targets in organs that move with respiration, e.g., liver tumors. For hepatocellular carcinoma (HCC), however, surgical treatment (resection, transplantation) is most likely to produce long-term survival; for non-resectable tumors, therapies other than radiosurgery are typically recommended. This study evaluated the long-lasting anti-tumor effects of CK combined with surgery in patients with HCC.

**Materials and methods:**

Eighteen patients (three women, 15 men) were included in this prospective observational study. They received 21 single-fraction CK treatments (26 Gy). Patient characteristics, treatment effects, tumor response (according to the Response Evaluation Criteria In Solid Tumors (RECIST) grading) and survival were measured for a median period of 29 months.

**Results:**

Local tumor control was achieved in 15 patients, with complete and partial remission observed in 10 and five patients, respectively. One patient was treated for two separate lesions in one session, and one received three treatments each separated by two-year intervals; both patients are tumor-free. Two patients showed minimal response, and in one patient local tumor viability could not be excluded by MRI. Nine patients had HCC recurrence, all distant to the treated site. Nine patients died during follow-up, including two with clear relation to tumor progress. Tumor-free survival was 79.4% after one year and 29.8% after three years, and the corresponding overall survival was 84.8% and 66%.

**Conclusion:**

This study shows the high effectiveness of single-session frameless CyberKnife radiosurgery for treatment of hepatocellular carcinoma and reconfirms previous results of fractioned radiotherapy of HCC. It also demonstrates the potential of radiosurgery to be combined with surgical concepts.

## Introduction

Patients with primary liver tumors, such as hepatocellular carcinoma (HCC), have several treatment options according to guideline-based recommendations [1-2]. Anatomical surgical resection is considered to offer the best long-term prognosis for tumor-free and overall survival for patients not eligible for liver transplantation [3]. However, surgery in patients with liver cirrhosis, the usual etiology for HCC, is associated with considerable risk of perioperative morbidity and mortality [4-6]. Alternatively, radiofrequency ablation, transarterial embolization, and radiotherapy are locally ablative strategies allowing local tumor control for smaller tumors [7]. In recent years, stereotactic body radiotherapy (SBRT) [8-9] evolved to become a new therapeutic tool for oligometastases and primary malignancies. This technique allows for effective ablation of malignant liver tumors without clinically relevant radiation exposure of surrounding tissue [10], thereby reducing the risk for radiation-induced liver disease (RILD) [10-11]. Studies from Japan with large patient populations examined the feasibility and effectiveness of conventional SBRT with fractionated doses between 40-60 Gy in 4-10 sessions. Survival rates of 53% with a median follow-up of 68.3 months were obtained for patients with HCC [12]. In the same study, disease-free and distant metastases-free survival reached 39.9% and 76.3%, respectively [12]. However, SBRT delivered with conventional linear accelerators is far from being an ideal treatment method. Complex immobilization devices are typically required to suppress respiratory motion of the liver so that high doses can be effectively targeted to the tumor while sparing normal tissues [8,12]. The therapeutic concept of radiosurgery was initially designed by Lars Leksell, a Swedish neurosurgeon, half a century ago [13]. In this first version (now called the Gamma Knife), a coordinate system in the form of a calibrated metal frame was attached to the patient's head, enabling immobilization and precise targeting (within 0.5 mm) of the region of interest. This allowed only treatment of the brain and upper spine in a single-session radiosurgical approach. More recently computer-assisted frameless radiosurgical systems have emerged that allow the extension of radiosurgery outside the brain, such as to the lung or liver [14-17].

In this respect, the CyberKnife System (CK; Accuracy Inc., Sunnyvale, California, U.S.A.) allows for a much more focused stereotactic approach based on real-time tracking of the target, and thus represents the next step towards a minimally invasive, individualized radiation treatment. It enables the precise application of a high (tumoricidal) radiation dose to a well-defined target volume (convergent beam irradiation) while sparing the surrounding healthy structures in a single session without fixation devices [14-16]. Such a technique could serve as an ideal add-on tool for surgical therapies, e.g., “bridging-to-transplant,” or within a multimodal ablative concept in multifocal or bilobar HCCs. These strategies could combine the surgical resection of the dominant lesion and CyberKnife for elimination of smaller additional lesions in the liver remnant.

To date promising studies reporting on CK radiosurgery for liver tumors concentrate mostly on feasibility and peri-interventional results with short observation intervals [12, 18-20]. This prospective observational study seeks to provide insights into the midterm treatment effects of CK therapy on adverse events, tumor control and patient survival, and its potential to serve as an adjunct to liver surgery in HCC patients.

## Materials and methods

**Figure 1:**
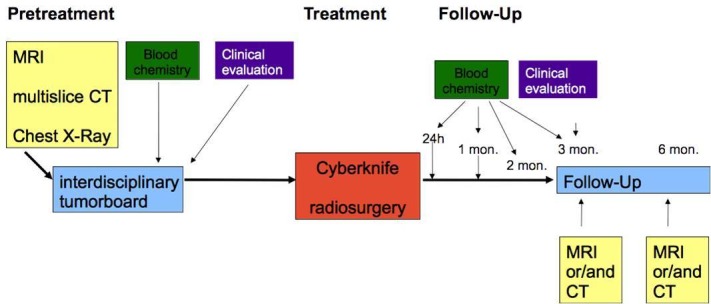
Treatment and follow-up algorithm as approved by the ethics committee.

At the University Hospital Munich, CyberKnife therapy for HCC was established in 2006. Since then, 21 separate treatments in 18 patients were undertaken. The diagnosis of hepatocellular carcinoma was based on either (a) biopsy or (b) characteristic contrast patterns in two complementary slice imaging techniques (i.e., CT and MRI). Patients were treated after consensus recommendation based on individual case presentations in the interdisciplinary tumor board. The before-treatment presentation of each case with HCC in this tumor board is a substantial element of the “Comprehensive Cancer Center” at our institution. At the respective stages, interdisciplinary stratification between liver transplantation, surgical resection, radiofrequency ablation, systemic treatment as standard treatments (as recommended by the German S3-Guidelines for HCC treatment), and individual innovative treatments such as CyberKnife are conducted. Once CyberKnife treatment was chosen, all patients were monitored for a minimum of 12 months according to a strict algorithm for follow-up examinations (Figure 1). 

Follow-up was based on continuously updated patient charts of our hospital, supplemented by a questionnaire sent to the primary care physicians. This allowed for a completion of the data records in all cases, including when patients missed scheduled follow-up appointments. Response to radiosurgery was expressed as complete remission (CR), partial remission (PR), minor remission (MR), no change (NC), and progressive disease (PD) based on post-interventional MRI imaging, according to the Response Evaluation Criteria In Solid Tumors (RECIST) [21]. Clearly viable tumor residuum or recurrence was defined as treatment failure.

The study was performed in complete agreement with GCP guidelines for retrospective analyses. This study was approved by the ethics committee of the University of Munich (ethics grant no. 058-11).

### CyberKnife technique

Patient Preparation

For the treatment of liver tumors, one to three small (5 mm) gold fiducials were percutaneously placed in the area of the tumor under computed tomography (CT) guidance using a 128 detector scanner (Siemens, Erlangen, Germany). In addition, magnetic resonance imaging was performed in order to facilitate tumor delineation. The implanted fiducials served as landmarks for real-time tumor tracking and were automatically detected by the stereo digital X-ray camera system of the CyberKnife. Tumor delineation, the organs at risk, the target dose to the tumor, and the tolerance doses of the organs at risk were determined by the attending physician. All lesions were treated to 26 Gy (70% isodose) to a 5-mm margin around the lesion.

Radiation System

The CyberKnife technology is intended to accurately deliver multiple beams from wide-ranging angles to treatment targets throughout the body. A lightweight and compact photon emitter (6 MeV LINAC dose rate 6 Gy / minute) is mounted on a six-jointed robot arm (Kuka GmbH, Augsburg, Germany). Typically, 100 to 150 circular beams of varying diameters are used for each treatment session. The robotic system is connected to a computer-controlled positioning system. Before treatment, the patient is positioned so that the target region (i.e., tumor) is in the middle of the image-guidance system, which enables precise target localization automatically throughout the session. Orthogonal X-rays are registered to digitally reconstructed radiographs derived from the treatment planning CT-scan data. Offsets between the current digital images and the pre-treatment durable response rates (DRRs) inform the treatment system of the current location of the target—deviations within 10 mm and 5° of the intended position are corrected by adjusting the robot’s aim of the treatment beam, and larger deviations require the patient to be re-positioned.

Dynamic Position Correction

To treat a target volume that moves with respiration, fiducials implanted near the tumor are imaged periodically and their position is correlated with the position of optical signals (LEDs) on the patient’s chest. Various linear and non-linear correlation models are fitted to the obtained locations to maximize the predictability of the internal target position based on the external signal location. The correlation allows the aim of the robot to dynamically track the internal target based on the motion of the external signals [15]. The correlation is updated throughout treatment with each set of internal images, allowing dose-delivery accuracy within a millimeter [16].

Statistical Analysis

Relations of patient and treatment characteristics were evaluated using the procedures 'descriptives,' 'crosstabs,' 'chi-square' (Fisher's exact test for low case numbers) using SPSS (version 23.0, IBM Software, New York, U.S.A.) as appropriate. Survival analysis was performed using the Kaplan-Meier method.

## Results

### General data

**Figure 2:**
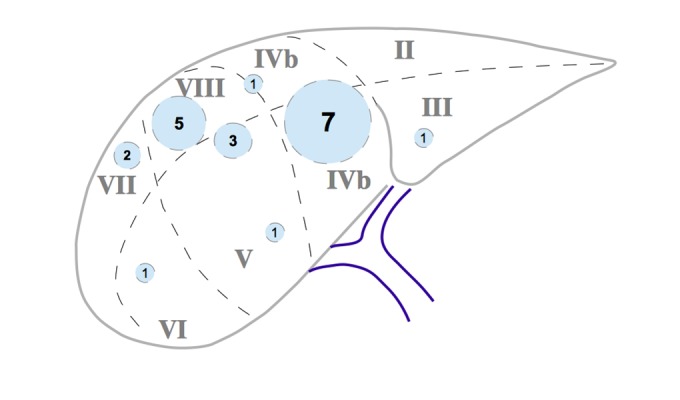
Intrahepatic distribution of the treated hepatocellular carcinoma lesions.

Eighteen patients, three women and 15 men, with the diagnosis of HCC were treated with CK as recommended by the interdisciplinary tumor board. Tumors were localized in central liver segments in the majority of the patients (Figure 2).

Mean age was 70.8 years (median 72 years) (Table 1).

**Table 1:** General patient characteristics

Parameter	Number or Mean ± SEM; Median (Range)
Gender men / women (n)	15 men / 3 women
Interventions in men / women (n)	16 men / 5 women
Age (years)	70.8 ± 11.0; 72 (48-92)
Tumor diameter (CM)	2.9 ± 0.42; 2.6 (1.0-8.2)
AFP (ng/dl)	77.4 ± 66.1; 5.8 (1-1200)
AFP elevation (n)	7

Two men were treated for two different tumors during the same session and one woman was treated three times for three different lesions sequentially. One woman and three men had surgery prior to radiosurgery. 

**Figure 3:**
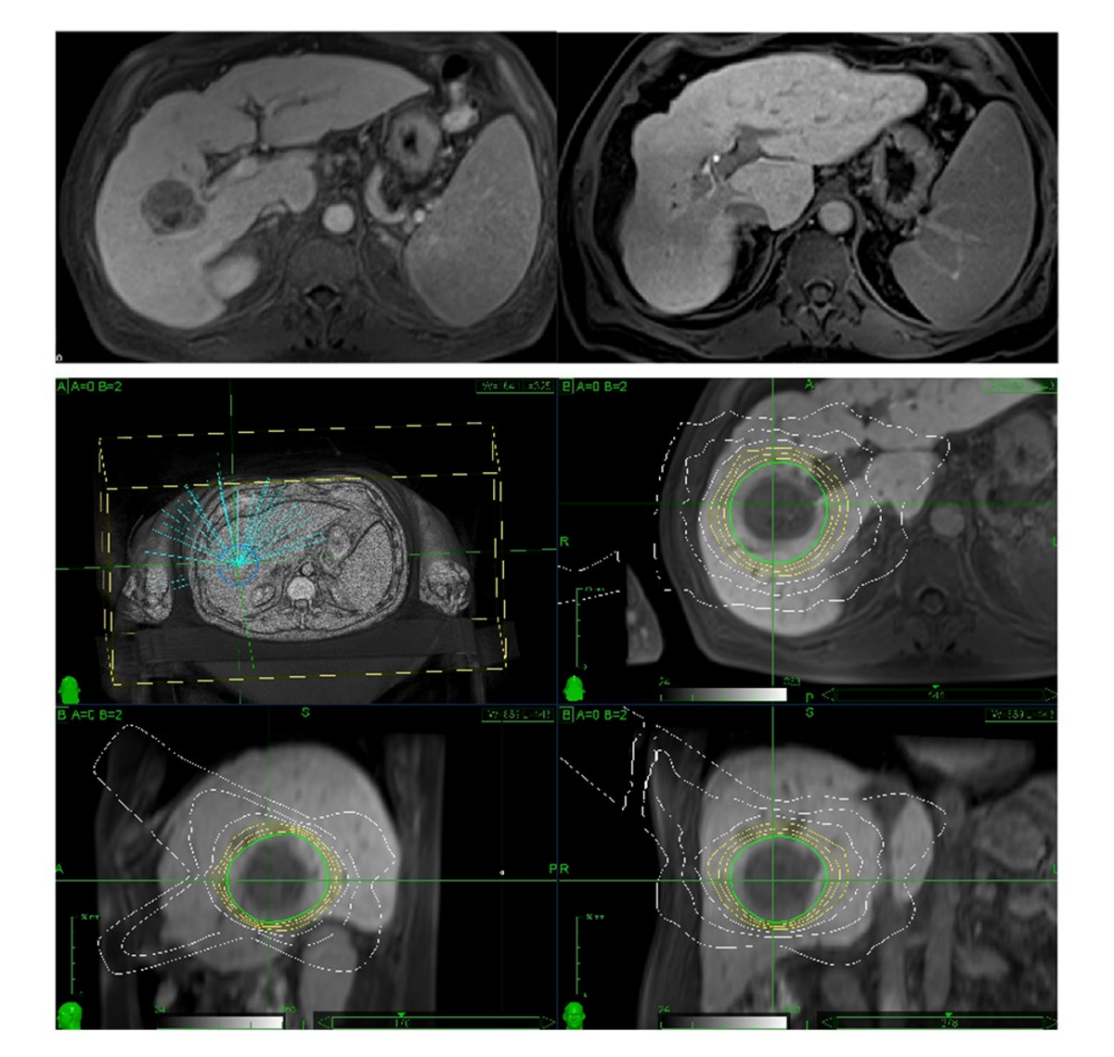
Treatment of a centrally located hepatocellular carcinoma in segment 7 in a patient with Child A cirrhosis. Imaging before and after CK radiosurgery (above) and prescription planning (below).

In all cases, alternative treatment options were considered but were deemed likely to be less effective or to pose a risk, mainly due to tumor locations requiring extended resections to achieve complete HCC removal (Figure 3).

All patients revealed clear morphological signs of structural liver parenchyma injury by cirrhosis or fibrosis. Proven liver cirrhosis Child A (12) or B (five) was present in all patients, one patient was considered to have liver fibrosis based on clinical chemistry results without radiomorphological signs of cirrhosis. The underlying diseases were hepatitis C (six), hepatitis B (four), hepatic steatosis (five) and alcohol abuse (six) (Table 2).

**Table 2:** Pre-Interventional prerequisites

Pre-Interventional Prerequisites	
Cirrhosis	
Child A	12
Child B	5
Fibrosis	1
Adipositas	9
Cardio-pulmonary preexisting disease	7
> 1 lesion	4
Pre-interventional treatment	
Surgery	4
TACE	4
Radiofrequency ablation	3
Radiation	1 (2x CK); 1 (fractionated SBRT)

This was accompanied by elevated serum levels of enzymes and reduced clotting factors indicating disturbed hepatic function (Table 3).

**Table 3:** Preinterventional laboratory parameters

Laboratory Parameter	Mean ± SEM; Median (Range)	
AP (U/l)	163.7 ± 34.6; 132 (27-643)	
Gamma GT (U/l)	172.5 ± 42.5; 134 (19-683)	
Bilirubine (mg/dl)	2.3 ± 0.66; 1.1 (0.4-10.8)	
GOT (U/l)	80.7 ± 11.6; 64 (30-175)	
GPT (U/l)	69.5 ± 13.5; (188-240)	

Increased cardiopulmonary risk was evident in seven patients. Together these factors led to the decision not to perform surgical resection.

### HCC characteristics

Mean alpha-feto protein (AFP) was 77.4 ± 66.1 U/ml (median 5.75, range 1199 U/l) which was elevated in seven patients. The diameter of the lesion treated was 2.9 ± 0.4 cm (median 2.6 cm, range 1.0 to 8.2 cm) (Table 1).

### Tumor response and side effects

Local tumor control was achieved in all but one patient after a mean follow-up of 29.6 (median 29) months. Ten patients showed complete remission (CR), among them were the two patients with treatment of more than one tumor. In these two patients all treatments (n=5) lead to complete remission. Five patients exhibited partial (PR) and two minimal response (MR). In one patient no change (NC) was observed, whereas one showed some signs of local tumor viability (Eovist® contrast behavior) without tumor enlargement on follow-up MRI. Two patients had mild adverse reactions such as nausea and required no special treatment. No peri-interventional complications or the necessity to switch therapy was observed, and no patient experienced complications due to fiducial implantation.

One patient underwent surgery to treat tumor recurrence with two foci distant to the area treated by CK by bi-segmental resection and lymphonodectomy. He developed peritoneal carcinosis and diffuse intrahepatic HCC spread and died under medical and best supportive care two years after CK treatment. 

### Survival

**Figure 4:**
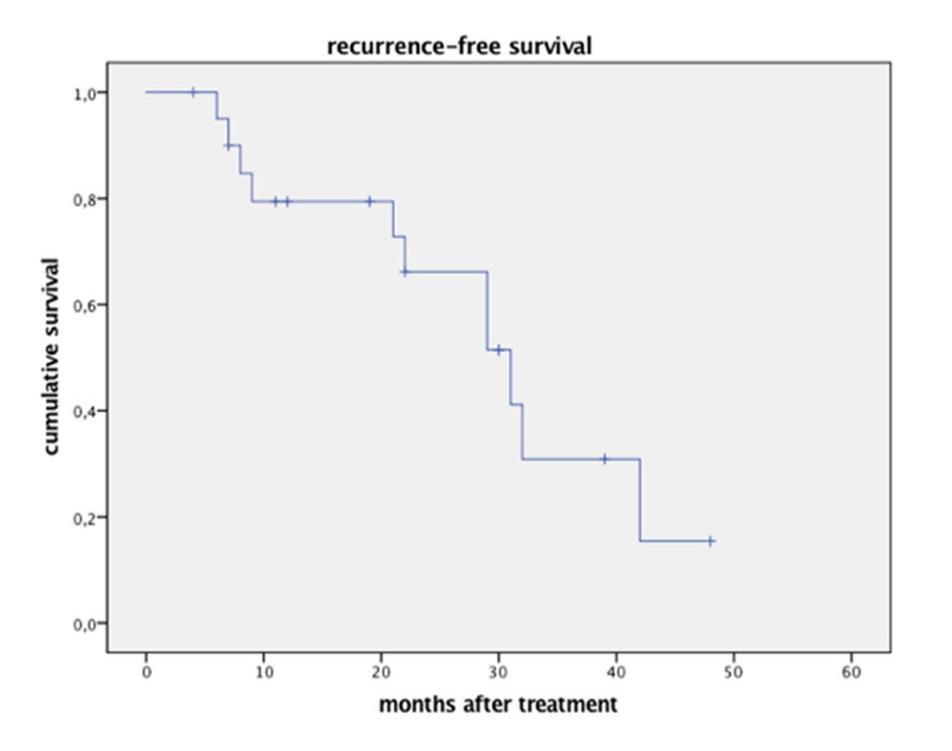
Disease-free survival after single-session CK radiosurgery (note that the two patients with repeat treatment are listed as separate events).

Mean recurrence-free survival was 21.8 months (median 21.0, range 6-48 months), with a local recurrence in one patient after 20 months. Twelve patients presented tumor recurrence distant to the treated site (extrahepatic or in other liver segments) during the complete observation interval. This resulted in tumor-free survival of 79.4% after one year, 29.8% after three years, and 14.9% after 42 months (Figure 4).

**Figure 5:**
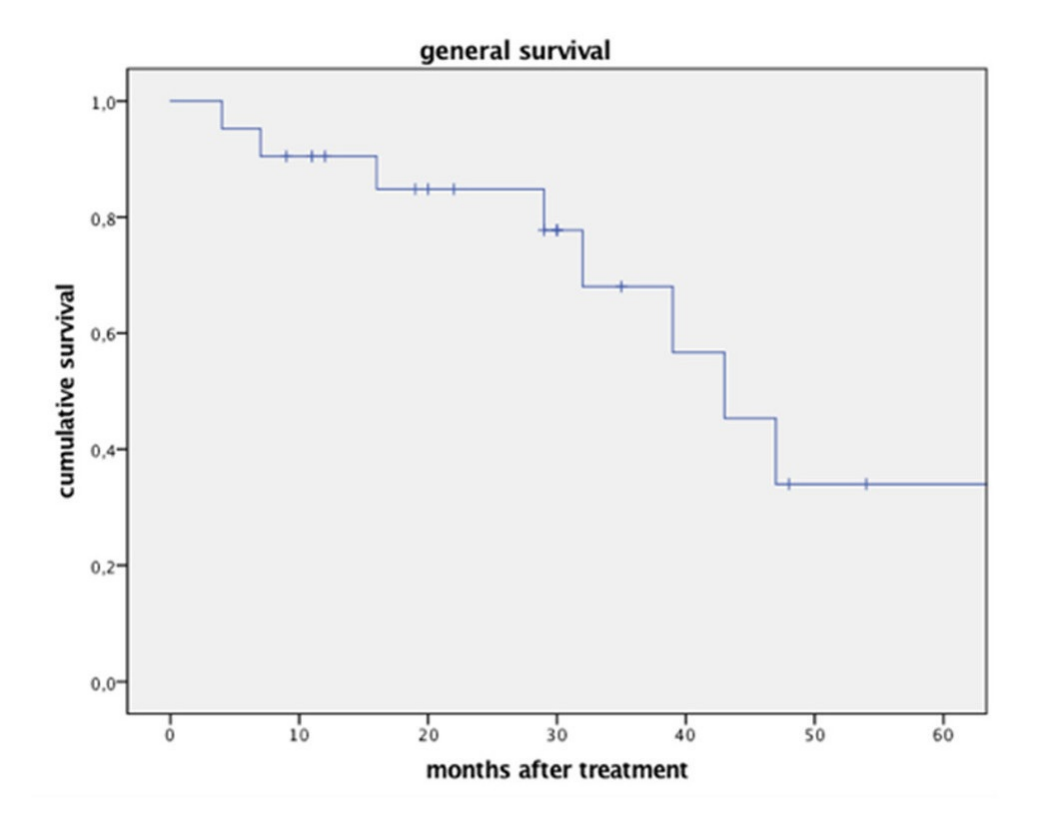
Overall survival after single-session CK radiosurgery.

Due to other adjuvant treatments the high distant HCC recurrence ratio did not translate into general survival, which was 84.8% after one year, 66% after three years and 33% after five years (Figure 5).

## Discussion

This study reports on mid-term results of a single center’s experience treating patients suffering from HCC with CyberKnife radiosurgery. Effective treatment with CK of liver metastases from colorectal carcinoma has been published by our institution before [22-23]. The present cohort of patients with HCC underwent a similar single-session CK treatment under outpatient conditions. Before treatment all patients were discussed in our interdisciplinary tumor board. CyberKnife radiosurgery was considered as the appropriate regimen to treat the HCC lesions after ruling out the established standard treatment options including surgical resection, liver transplantation, radiofrequency ablation (RFA), transarterial chemoembolization (TACE) or systemic treatment (such as sorafenib). Because of this rigorous selection process, the patient cohort is rather small, with 21 lesions treated in 18 patients.

Our data show that a median tumor-free interval of 20 months may be expected after CK therapy. Local recurrence was only noted in one patient (5.56%). Consistent with the likely systemic nature of HCC, recurrence distant to the treated foci was observed in twelve patients. In contrast with other published studies, applying fractionated CK radiation [14,18], single-session CK radiosurgery to a dose of 26 Gy resulted in only one local recurrence. Although the cumulative risk of recurrence in our patients was higher due to the longer follow-up period, the local recurrence rate was markedly lower in our cohort. These previous studies reported local tumor control rates of 55% and 77% after CK treatment with 28-60 (median 40) Gy in four to five fractions, respectively [18,20]. In addition, our results were closer to a study from Japan, using conventional SBRT in 79 patients, which showed 80% of patients without local tumor progression [12]. The high local tumor control rate may reflect the accuracy (to within about 1.0 mm) of the CyberKnife system. Additionally, in contrast to our analysis, the average tumor diameter in the studies by Lo et al. and Que et al. was larger; 58% of tumors were larger than 5 cm in the report by Lo et al., and 100% were larger than 10 cm in the study by Que et al.. Besides this, also the lower ratios of patients with hepatitis B or C, in Child B and C cirrhosis stages, and less macro-vascular portal vein involvement for patients in our study may have contributed to the observed effect [18,20]. In contrast, the patients in the current study were followed for a longer period, thus exposing them to a higher risk of local recurrence. Also, tumor response rate has been related to total treatment dose [24-25]. We used only a single-fraction dose of 26 Gy to the tumor margin, giving a central dose of over 30 Gy.

The dose effect on tumor biology of single session radiation, where the complete dose is applied at once, is difficult to predict. The LQ-model has been developed to anticipate effects of low dose per fraction radiation as applied in conventional hyper- or hypofractionated regimen. Compared to previous observations after hyperfractionted SBRT [26-27] the current work demonstrates higher rates of local tumor control.

From a surgeon’s point of view, our observations have two implications. Surgical resection or liver transplantation are preferred because the 5-year disease-free survival approximates 15-55% after resection and 75% after transplantation [3,28]. Nevertheless, not all patients are eligible for these strategies at the time of the diagnosis. Complex or extended liver resections may be dangerous or impossible in the presence of liver cirrhosis, even when classified as Child A. With CK radiosurgery as a presurgical therapy the tumors can obviously remain well controlled until extended resections with preconditioning concepts can be planned and realized as definitive treatment [29].

Local control of HCC lesions by CK radiosurgery after resection may be useful to overcome the risk of tumor growth stimulation during hepatic regeneration [30]. In addition, in situations of multifocal intrahepatic distribution of HCC lesions, centrally located lesions (in liver segments 4a, 4b, 5 and 8) might be sufficiently treated by CK radiosurgery, thus allowing the surgical resection of peripherally located HCC lesions. This concept of combining locally ablative strategies with other treatment modalities in primary liver cancer has been described by others [31] and is basically a splitting of the therapies with the aim of complete tumor eradication. However, Liu and coworkers did not describe hepatic resection combined with radiotherapeutic approaches [31-32]. Taken a step further, a local tumor control strategy by CK radiosurgery could be considered as a simultaneous therapeutic approach for patients who require portal venous embolization (PVE) to stimulate hypertrophy of the liver remnant. Alternatively, it might be combined with the in situ splitting strategy of two-stage hepatic resection [33]. Together with these surgical strategies, CK could safely facilitate hypertrophy of the remnant liver, thus representing an effective treatment alternative for patients with bilateral HCC, in whom radiofrequency ablation or TACE (perhaps intraoperative) represented the therapeutic limit until now. In our study population, one patient successfully underwent liver transplantation after achieving local control over his HCC. Another patient had a liver resection of liver segments 6 and 7 for two HCC lesions after having treated a centrally located third lesion centrally in segment 8. The procedure was well tolerated and he was free of any malignancy, whereas an extension of the resection—a trisectorectomy—in this patient to treat all lesions by surgery was considered to override the tolerable limit of liver tissue loss.

Another complementary use of radiosurgery and surgery has been called “bridging to transplant.” The effectiveness of fractionated radiotherapy (SBRT) to treat HCC patients on the waiting list for liver transplantation has been published [19]. Some patients appear to be exposed to a high peri-interventional risk by applying RFA or TACE before liver transplantation, due to their reduced synthetic and metabolic hepatic function in combination with ascites [34]. These patients would benefit from the non-invasive treatment by CK, particularly, if fiducial-free strategies are expanded to be used in liver treatments [35]. 

According to German guidelines for HCC treatment [2], the standard local treatment technique for single HCCs in patients not eligible for surgery or transplantation is RFA. However, RFA is associated with some hospitalization, due to the setup of the treatment algorithm and the necessity of surveillance for a minimum of 24 hours after RFA. In addition, in non-selected patient cohorts, complete surgical resection of HCCs still seems to offer the best long-term results of survival besides liver transplantation [3,34]. Data comparing surgery to locally ablative treatments shows clearly better local tumor control and long-term survival for surgery [3,34]. Data from small samples of highly selected patients comparing CK and surgical excision for stage 1 HCC [36] or with portal vein thrombosis [32] show comparable overall survival [36]. Although comparisons between CK, RFA or surgical methods were beyond the scope of this study, we were able to show that repeated treatment represents an option for patients with HCC recurrence. Taking all these factors into consideration, we propose that the spectrum of options for local HCC control is widened by the addition of CK radiosurgery.

### CyberKnife radiosurgery or SBRT – what should a surgeon know?

Although there is more data available on SBRT for liver lesions, some advantages of CyberKnife radiosurgery should be discussed. The local tumor control rate is dependent on the precision of the applied dose targeted on the malignant lesion. Real time fiducial tracking with interfraction corrections enables a very precise targeting of the lesion and thereby a reduced tumor margin [3,34]. It has been shown, that conventional radiation therapy with prescribed fractionated doses between 50 to 70 Gy failed to prevent local HCC recurrence [37]. The low local recurrence rate observed in our study compared to others also challenges the presumption that higher cumulative radiation doses are biologically more effective in local HCC eradication. Additionally increasing radiation intensities and radiation field sizes (necessary in the absence of real-time tracking and correction of beam aim with respiration), may also increase the rate of side effects [38]. The percentage of gastrointestinal toxicity after SBRT reached 7 to 8.3% in larger studies [39-40]. Hepatic adverse reactions ("veno-occlusive disease," biliary obstruction) are expected in about 10% of patients after SBRT, especially in the presence of pre-existing parenchymal disturbances such as cirrhosis, hepatitis or chemotherapy effects [25, 41-42]. CK radiosurgery in the present study revealed a much lower rate of adverse effects (2/18 (11%) patients with grade 1, which may reflect the fact that targeting precision of the CK allows treatment of the tumor with a much smaller margin to account to error, and thus results in exposure of normal liver tissue to less high-dose radiation. These factors would favor CK as a tool in multimodal treatment concepts involving surgery. 

It is also of tremendous practical significance that fractionated radiotherapy and SBRT both require a longer time to deliver than single-fraction radiosurgery (a fact which can greatly impact a patient’s quality of life). Particularly in the context of in-situ splitting with surgical resection or PVE, the time loss associated with fractionated radiotherapy may result in surgery being postponed. Conventional SBRT strategies are typically accomplished by immobilizing patients using body frames, vacuum pillows, or abdominal compression to limit respiratory motion of treated tumors [8-9, 43-44]. The CK requires no such immobilization, and as such may be much more comfortable for patients.

## Conclusions

With the latest technological development, the spectrum of indications of radiosurgery can be transferred to visceral organs such as the liver. Although the case number in this single-center study only allows for a limited interpretation, it shows that CK offers a safe and effective frameless radiosurgical treatment for HCC, with tumor control comparable to the therapeutic standard of radiofrequency ablation. This new indication for radiosurgery may be ideal for patients who are no longer eligible for surgical or other alternative therapies due to their general clinical situation or comorbidities. Additionally, from a surgeon’s standpoint, CK radiosurgery may be an interesting tool to support definitive treatment via surgical resection or transplantation. We believe interdisciplinary tumor conferences in which all available treatment options are discussed are important for confident patient selection and stratification before radiosurgery is recommended.
